# Proteomic Signatures and an Injury-Stress Endotype in Myositis-Associated Interstitial Lung Disease

**DOI:** 10.64898/2026.08.04.26359441

**Published:** 2026-08-06

**Authors:** Julio A. Huapaya, Peter D. Burbelo, Eric W. Robbins, Xin Tian, Shijinqiu Gao, Sevilay Turan, Salina Gairhe, James M. Ward, Neelam Redekar, Jianliang Li, Gloria Pastor, Neha A. Gupta, Payam Noroozi Farhadi, Kakali Sarkar, Maria Casal-Dominguez, Iago Pinal-Fernandez, Lisa Christopher-Stine, Adam Schiffenbauer, Lisa G. Rider, Andrew L. Mammen, Sonye K. Danoff, Anthony F. Suffredini

## Abstract

**Rationale:**

Idiopathic inflammatory myopathy-associated interstitial lung disease (IIM-ILD) is a major cause of morbidity and mortality. We tested whether quantitative myositis-specific autoantibodies and proteomic profiling capture biological heterogeneity and prognosis beyond categorical serology.

**Methods:**

Myositis-specific autoantibodies were quantified using the luciferase immunoprecipitation systems assay, and 184 serum proteins were measured in 226 IIM patients; 199 with higher-ILD-risk autoantibodies (Jo-1/MDA5/PL-7/PL-12/EJ), 27 with lower- ILD-risk autoantibodies (Mi-2/NXP2/TIF1γ) and 35 healthy controls. We identified shared and subgroup-specific differences by comparing each subgroup with controls, then correlated quantitative autoantibody and protein levels within higher-risk subgroups. Additional analyses included pathway enrichment, unsupervised clustering, longitudinal lung-function change, and mortality.

**Results:**

Higher-ILD-risk subgroups shared interferon-responsive CXCR3 chemokine, IL- 6/JAK/STAT3, and apoptosis signaling. Dominant autoantibody subgroup profiles differed: interferon/CXCR3 chemokine signaling with T-cell activation and monocyte recruitment in anti- Jo-1; proteostasis/antigen-processing and vascular/cellular stress signals in anti-MDA5; IL- 6/macrophage and profibrotic signals in anti-PL-12; and apoptotic and innate immune activation with metabolic/redox-stress signals in anti-PL-7. Within higher-ILD-risk subgroups, autoantibody levels correlated with interferon-response, profibrotic, and metabolic/vascular proteins (r=0.40- 0.74; nominal p<0.05). Unsupervised clustering identified four proteomic endotypes beyond autoantibody type, including an injury-stress endotype associated with worse lung function and poorer survival, and a chemokine/checkpoint-high endotype with relatively preserved lung function. Across 203 participants with 38 deaths, a weighted 10-protein score was associated with all-cause mortality (HR, 3.28; 95% CI, 2.12-5.08; p<0.001).

**Conclusions:**

Integrated quantitative autoantibodies and proteomic profiling revealed shared inflammatory biology, autoantibody-associated signatures, and an injury-stress endotype associated with poor survival in IIM-ILD, supporting risk stratification beyond categorical serology.

## Full Text Availability

The license terms selected by the author(s) for this preprint version do not permit archiving in PMC. The full text is available from the preprint server.

